# Professor James H. Conway

**Published:** 1865-02

**Authors:** 


### OBITUARIES.

### *Professor James H. Conway.*

We record, with feelings of profound regret, the untimely death of James H. Conway, M. D., Professor of Obstetrics and Diseases of Women and Children in the Medical College of Virginia, and one of the most eminent physicians of Richmond.

Friendship finds an easy task in rehearsing the many admirable qualities of head and heart which characterized this gentleman. Endowed by nature with a good intellect, an attractive person, and a sweet and genial temper, he was likewise remarkable for peculiar graces of manner and carriage which combined to render him an universal favorite, and gave him a circle of friends and admirers who showered on his path through life their favors and good offices.

Thus gifted by nature and education, success in his profession was the necessary consequence, and constant study and observation only aided his rapid progress to the higher ranks of his chosen calling. Hence, when quite young, he was a lecturer on obstetrics in the summer session of the Medical College of Virginia, and on the death of Professor Bohannon he was appointed to his vacant chair, where he exhibited a close and careful knowledge of its important requirements, which rendered him a most useful and industrious instructor

His death at an early age—ere he had reached his period of greatest usefulness, has deprived the medical circle of Richmond of one of its chief ornaments. Society mourns his loss, and he leaves not an enemy behind.

